# The complete chloroplast genome of *Melilotus albus*: an important source of forage species

**DOI:** 10.1080/23802359.2018.1473730

**Published:** 2018-05-15

**Authors:** Shicui Jiang, Long-Yue Meng, Junfeng Zhai

**Affiliations:** a MOE Key Laboratory of Natural Resources of the Changbai Mountain and Functional Molecules, Yanbian University, Yanji, China;; b Chinese Academy of Inspection and Quarantine, Beijing, China

**Keywords:** *Melilotus albus*; *Medicago*, chloroplast genome, phylogenetic relationship

## Abstract

*Melilotus albus* belongs to the legume family. It is known as the greatest potential forage crop and native to Eurasia and North Africa. The nitrogen fixation rate of *Melilotus albus* is superior to other legumes, making it beneficial for crop rotations. *Melilotus albus* has also been a species with good forage productivity and is more and more important in the medicinal value of its high coumarin content. In this study, the complete chloroplast genome of *Melilotus albus* was assembled and analysed. The complete chloroplast genome of *Melilotus albus* was 127,205 bp in length. It harbours 108 functional genes, including 76 protein-coding genes, 28 transfer RNA, and four ribosomal RNA genes species. The overall nucleotide composition was: 33.3% A, 33.1% T, 16.3% C, and 17.3% G, with a total A + T rich content of 66.4%. Phylogenetic relationship analysis showed that *Melilotus albus* has closely related to *Medicago.*

*Melilotus albus* is one of the most important legume forages and is also recognized as an important source of forage for ruminant animals in rangeland (Stevenson 1969). It has been adapted to extreme environments, such as drought, cold, and saline areas. The nitrogen fixation rate of *Melilotus albus* is superior to other legumes, making it beneficial for crop rotations. It has been also a species with good forage productivity and is more and more important in the medicinal value of its high coumarin content (Luo et al. 2017). In this study, we get the complete chloroplast genome of *M. albus*, in order to provide information for the study of the origin of forage crop in evolution.

The specimen of *Melilotus albus* was isolated from Yanbian University test field in Yanji, Jilin, China (129.48E; 42.91N) and the DNA of *M. albus* was stored in Yanbian University (No. YBU01). The *M. albus* DNA sample was sequenced by the Illumina HiSeq4000 system manufacturer instructions. The *M. albus* complete chloroplast genome was preliminarily annotated using the DOGMA (Dual Organellar GenoMe Annotator) online program (Wyman et al. 2004) and tRNAscan-SE (Lowe and Chan 2016).

The chloroplast genome sequence of *Melilotus albus* is a closed loop that is 127,205 bp length. It displayed 108 functional genes set which observed this plant chloroplast, including 76 PCGs, 28 tRNA genes (one for each amino acid, two each for methionine, valine, threonine, arginine, isoleucine, and serine, three each for leucine), and four genes for ribosomal RNA subunits (one for each *rrn*16, *rrn*23, *rrn*4.5, and *rrn*5). The annotated chloroplast genome had been submitted to GenBank database under accession no. MH191352.

For phylogenetic relationship analysis, we selected other 35 related complete chloroplast genomes from GenBank to assess the relationship between them. The genome-wide alignment of all plant complete chloroplast genomes was done by HomBlocks (Bi et al. 2017). The phylogenetic tree was reconstructed using the neighbor-joining (NJ) and maximum likelihood (ML) methods. ML analysis was performed using RaxML-8.2.4 (Stamatakis 2014), of which the bootstrap values were calculated using 5000 replicates to assess node support. NJ phylogenetic tree was constructed using MEGA7 with 5000 bootstrap replicates (Kumar et al. 2016). All the nodes were inferred with strong support by the ML and NJ methods, as shown in phylogenetic relationship analysis (Figure 1). *Melilotus albus* was the closest with *Medicago*.

**Figure 1.**
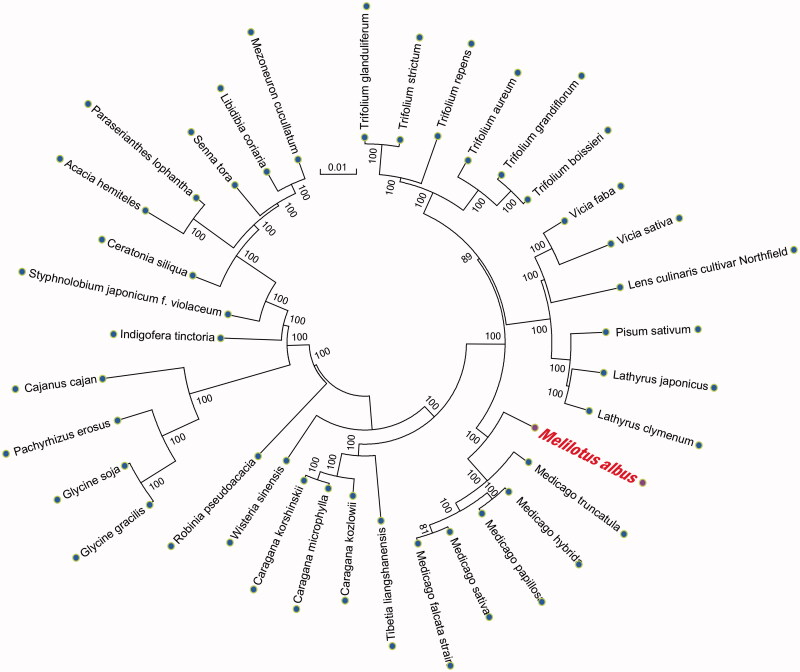
Phylogenetic relationships of *M. albus* and other 35 species were constructed based on plant complete chloroplast genomes using the neighbor-joining (NJ) and maximum likelihood (ML) with 5000 bootstrap replicates analysis. The tree was drawn without setting outgroups. Numbers in each of the node indicated the bootstrap support values.
